# Population genetic analyses are consistent with the introduction of *Ceramium secundatum* (Ceramiaceae, Rhodophyta) to Narragansett Bay, Rhode Island, USA

**DOI:** 10.1002/ece3.1754

**Published:** 2015-10-19

**Authors:** Meghann R. Bruce, Gary W. Saunders

**Affiliations:** ^1^ Department of BiologyCentre for Environmental and Molecular Algal ResearchUniversity of New Brunswick10 Bailey Dr.FrederictonNBCanadaE3B 5A3

**Keywords:** *Ceramium*, cox2‐3 spacer, DNA barcoding, introduced species

## Abstract

During ongoing DNA barcode (COI‐5P) surveys of the macroalgal flora along the northwest Atlantic coast, we discovered a population of *Ceramium secundatum* in Narragansett Bay, Rhode Island, USA. This species is regarded as common and widespread in the northeast Atlantic, ranging from Norway to Morocco, but until now has not been reported from the western Atlantic. Several lines of evidence suggest that *C. secundatum* may be introduced to Narragansett Bay: (1) despite extensive collecting, specimens have only been obtained from a limited geographic range in the northwest Atlantic; (2) three other nonindigenous seaweed species are reportedly introduced in this region, which is thought to be a consequence of shipping; and (3) this species is introduced to South Africa and New Zealand. To investigate this suspected introduction, we applied population genetic analyses (using the cox2‐3 spacer) to compare the Narragansett Bay *C. secundatum* population to native populations in the Republic of Ireland and the United Kingdom. Collectively, analyses of biogeographical and molecular data indicate that *C. secundatum* is likely introduced to Narragansett Bay. The implications of this discovery are discussed.

## Introduction

Monitoring of marine algal diversity is critical not only for understanding species richness across biogeographical ranges, but for detecting biological changes in the marine environment such as the introduction of nonindigenous species to novel regions. In recent studies, molecular tools have improved environmental monitoring, as well as facilitated the rapid detection of nonindigenous and potentially invasive species (e.g., Saunders 2009; Blanchet 2012; Savoie and Saunders 2013). In addition, molecular tools have helped to identify both the vicinity from which nonindigenous species have originated and, in turn, the potential vectors of introduction (e.g., Schaffelke et al. 2002; Provan et al. 2005; Blanchet 2012). Hence, the application of molecular tools has important implications for monitoring algal diversity and the establishment of marine conservation priorities.

While numerous molecular markers are useful for monitoring marine floras, the relatively recent application of DNA barcoding (COI‐5P surveys) has unquestionably made a significant contribution toward establishing accurate accounts of species diversity and distributions (e.g., Saunders 2005; Robba et al. 2006; Le Gall and Saunders 2010). At the Centre for Environmental and Molecular Algal Research, we are conducting ongoing COI‐5P surveys of the marine algal flora of Canada (e.g., Saunders 2008; Clarkston and Saunders 2012; Hind and Saunders 2013). The results of these COI‐5P surveys have not only provided regional inventories of macroalgae and furthered our knowledge of biogeographical distributions (e.g., McDevit and Saunders 2010), but they have also uncovered cryptic species complexes (e.g., Le Gall and Saunders 2010; Clarkston and Saunders 2012) and aided in the detection of introduced species (e.g., Saunders 2009; Savoie and Saunders 2013).

To assess whether a new macroalgal record for a particular region represents a taxon that was previously overlooked in floristic surveys or an introduced species, traditional approaches would recommend a careful review of relevant literature in addition to further morphological examinations of herbarium material in order to determine whether the species was previously collected and not reported, or incorrectly reported as a different species. Unfortunately, it is difficult if not impossible to identify and annotate historical herbarium material with certainty when specimens share similar morphological features. Even if morphology was useful to identify a species of interest in herbarium collections, this would only confirm that the species was previously collected, which fails to establish whether the species is native or introduced. Unlike traditional approaches, molecular‐assisted investigations are not hindered by the previously mentioned complications associated with interpretations of macroalgal morphology.

As part a COI‐5P survey of the macroalgal flora along the northwest Atlantic coast, we have been investigating the red algal genus *Ceramium* Roth. Morphologically, species within this genus have uniseriate axes with intervals of irregularly arranged cortical cells that are either restricted to nodal regions, giving an overall banded appearance of the thallus, or completely ensheathing the internodal regions (Maggs and Hommersand 1993; Maggs et al. 2002; Gabrielson et al. 2006). Currently, there are over 180 species recognized for the genus *Ceramium*, which are widely distributed throughout subtidal and intertidal habitats around the world (Maggs and Hommersand 1993; Maggs et al. 2002; Sears 2002; Gabrielson et al. 2006; Guiry and Guiry 2014). However, due to the complications associated with morphological‐based species identifications and rampant phenotypic plasticity in this genus, the taxonomic status, as well as current understanding of biogeographical distributions for many reported species, is uncertain or problematic for many species (Maggs and Hommersand 1993; Maggs et al. 2002).

Our COI‐5P survey of *Ceramium* species present in the northwest Atlantic has led to the discovery of a “cryptic” species complex for what is currently reported as *Ceramium virgatum* Roth in this region (Gabrielson et al. 2006). Whereas morphology failed to differentiate these entities, further genetic analyses (*rbc*L) have permitted the assignment of one of these genetic groups to *Ceramium secundatum* Lyngbye, a new record for the Northwest Atlantic flora. In this study, we assess the presence of *C. secundatum* in the northwest Atlantic using population genetic tools to ascertain whether it is native or introduced to this region. To accomplish this, we employed the noncoding intergenic region between the cytochrome oxidase subunit 2 and 3 genes (the cox2‐3 spacer), a quickly evolving haploid (mitochondrial) genetic marker that has proven useful at the population level in red algae (e.g., Zuccarello et al. 1999), and in exploring other species introductions (Andreakis et al. 2007). We hypothesized that if the population of *C. secundatum* to Narragansett Bay was a consequence of an introduction than this population should exhibit genetic characteristics resembling a founder event (Zink et al. 2000; Wares and Cunningham 2001). By comparing the Narragansett Bay population to populations within the native range, we expect that if *C. secundatum* is introduced we should observe the following: (1) lower genetic diversity within the Narragansett Bay population than comparable populations within this species' native range; and (2) haplotypes within the Narragansett Bay population represent a subset of those present within the native population (Zink et al. 2000; Wares and Cunningham 2001).

## Materials and Methods

### Collections

As part of an ongoing floristic survey (COI‐5P), 188 specimens assignable to the “morphospecies” *C. virgatum* were collected from the northwest Atlantic coast (Table S1, Supporting information). Subsequently, additional specimens of *C. secundatum* were collected from Narragansett Bay and four sites within the reported native range (within the Republic of Ireland and the United Kingdom) for a total of 91 individuals to be included in population genetic analyses (Table S2. Supporting information). In the field, specimens were collected from subtidal environments by SCUBA, snorkeling, wading at low tide or from pools in the lower intertidal. All specimens were dried and preserved as vouchers, either as a herbarium press or in a vial of silica, and a subsample of material was taken for DNA sequencing (Saunders and McDevit 2012).

### DNA sequencing

For the COI‐5P survey, DNA extraction was conducted according to Saunders and McDevit (2012). For COI‐5P, the PCR amplification profile followed Hebert et al. (2003) using primer combinations according to Saunders and Moore (2013). For taxonomic comparisons to data in GenBank, we generated *rbc*L sequence data for representatives of each resulting COI‐5P genetic group (Table S1, Supporting information; Saunders and Moore 2013). To compare genetic variation and haplotype diversity within the Narragansett population of *C. secundatum* to populations in the native range, we employed the quickly evolving mitochondrial spacer cox2‐3 (Zuccarello et al. 1999). For amplification of the cox 2‐3 spacer, we followed Zuccarello et al. (1999) using primers cox2for (Zuccarello et al. 1999) and cox3R (Gabrielson et al. 2002). Amplification products were sent to Genome Quebec for sequencing. Geneious version R6.0 (Biomatters, available from http://www.geneious.com) (Drummond et al. 2012) was used to edit raw data, generate contigs with complementary forward and reverse sequences, and align the resulting sequence data.

### Molecular population analyses

To compare genetic variation within and between populations, DNAsp (Librado and Rozas 2009) was used to measure: (1) number of polymorphic nucleotide sites, (2) average number of nucleotide differences (k), and (3) nucleotide diversity (*π*). To identify haplotypes, quantify their abundance within each population, and generate a 95% parsimony network, we used TCS (Templeton et al. 1992). EstimateS (Colwell et al. 2004) was used to conduct rarefaction analyses and investigate the probability of unsampled haplotypes within the native populations.

## Results

### Molecular‐assisted survey

While conducting a routine COI‐5P survey of the genus *Ceramium* in the northwest Atlantic, we identified a cryptic species complex for specimens that were morphologically attributable *C*.* virgatum* (Gabrielson et al. 2006). Analyses of *rbc*L sequence data established that one of the species uncovered was *C. secundatum* (*n* = 26) (GWS018041 (GenBank accession = KT250273) is a 100% match to GenBank accession #AF439287; Fig. 2A), the other genetic groups uncovered will be addressed in a future investigation. Minimum divergence (COI‐5P) between this species and nearest neighbor was 10.03%, with a maximum intraspecific variation of 0.31%. Although genetically distinct, *C. secundatum* was morphologically consistent with what is reported as *C. virgatum* for the northwest Atlantic (Sears 2002). All specimens were fully corticated with pseudodichotomous branching, commonly bearing numerous adventitious branches (Fig. 2). While *C. secundatum* specimens were collected from a limited geographical range (Narragansett Bay, Rhode Island with one drift specimen from Barnstable, Massachusetts; Table S1, Supporting information) and were almost exclusively subtidal (the exception being one tidepool collection), *C. virgatum* specimens were collected from numerous localities along the northwest Atlantic coast from both subtidal and intertidal habitats (Table S1, Supporting information).

### Population genetic analyses

**Figure 1:**
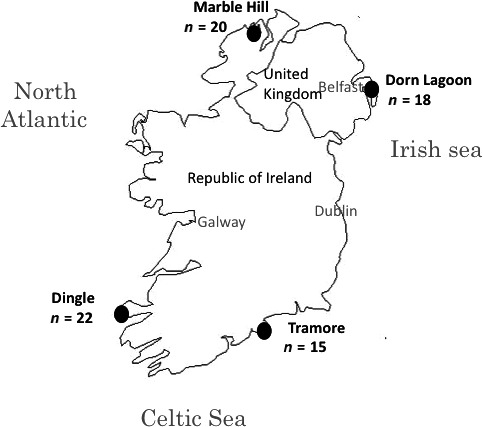
Sampled populations within the native range of *C. secundatum*.

We obtained cox2‐3 sequences (318 bp) for 16 of the *C. secundatum* specimens collected in Narragansett Bay. For specimens collected within the native range, we obtained cox2‐3 spacer sequences from 75 individuals in four populations: 22 from Dingle, 18 from Dorn Lagoon, 20 from Marble Hill, and 15 from Tramore (Table S2, Supporting information; Fig. 1). Overall, genetic variation within the Narragansett Bay population was lower than populations within the native range for all three measurements of variation obtained (Table 1). One exception to this is the number of polymorphic nucleotide sites in the Dorn Lagoon population, which matched the Narragansett Bay population (Table 1). Regardless, measurements of both k and *π* were still almost 2× higher in the Dorn Lagoon population than in Narragansett Bay (Table 1). In comparison with other populations within the native range, all three measurements of genetic variation were lower within the Dorn Lagoon population (Table 1).

**Table 1:** Measurements of genetic diversity within *C. secundatum* from Narragansett Bay in comparison with populations within the native range

	Population of interest	Populations within the native range			
	Narragansett Bay	Marble Hill	Dorn Lagoon	Tramore	Dingle
	*n* = 16	*n* = 20	*n* = 18	*n* = 15	*n* = 22
Number of polymorphic nucleotide sites	2	14	2	6	6
Average number of nucleotide differences (k)	0.525	2.568	0.954	2.857	2.736
Nucleotide diversity (*π*)	0.00165	0.00836	0.00300	0.00898	0.00860

Eleven haplotypes (HAP A‐K) were identified by our genetic analyses (Fig. 3; Table 2). The most widespread haplotype, HAP A, was present in all of the populations analyzed (Fig. 3; Table 2). The most abundant haplotype, HAP B, represented 34% of specimens included in our analyses, and it was also the second most widespread haplotype (Fig. 3; Table 2). Collectively, HAP A and HAP B comprised the majority (93.8%) of the haplotypes in the Narragansett Bay population. One haplotype, HAP C, was unique to the Narragansett Bay population and was not identified for any of the sampled populations from within the native range. Notably, all of the populations except Dingle had at least one haplotype that was unique (HAP D‐I and HAP K; Fig. 3; Table 2). Rarefaction analyses (Table S3, Supporting information) estimated upwards of 51 haplotypes within the sampled range, which suggests that HAP C may have been missed in our limited sampling of the native range.

**Table 2:** Distribution and abundance of cox2‐3 spacer haplotypes identified for all *C. secundatum* populations sampled

Population	Haplotype										
	A	B	C	D	E	F	G	H	I	J	K
Narragansett Bay	4	11	1	–	–	–	–	–	–	–	–
Marble Hill	1	–	–	8	1	8	1	1	–	–	–
Dorn Lagoon	4	9	–	–	–	–	–	–	5	–	–
Tramore	4	1	–	–	–	–	–	–	–	9	1
Dingle	4	10	–	–	–	–	–	–	–	8	–

Collectively, our results indicated that the genetic variation within the Narragansett Bay population of *C. secundatum* was lower than populations within the native range and that the haplotypes (with the exception of HAP C) were subsets of those within the native range, all consistent with the expected characteristics of a species introduction.

## Discussion

Introduced species have the potential to compromise native biodiversity, potentially endangering or further threatening rare species through the disruption of the ecological community balance (Walker and Kendrick 1998; Bax et al. 2003). This happens either through the slow process of genetic pollution, when introduced species hybridize with native species and replace locally adapted genotypes (Wares et al. 2005), or often more inconspicuously through competition with native species (Scheibling and Gagnon 2006). These negative impacts that transpire when introduced species become invasive create irreversible modifications to native communities (Teske et al. 2011). Cryptic introductions are particularly problematic because of the challenges in detecting, quantifying and monitoring changes within the ecosystem (Geller et al. 1997). Molecular‐assisted investigations are often necessary to overcome these biomonitoring challenges in order to identify cryptic taxa and assess putative introductions (Geoffroy et al. 2012).

**Figure 2:**
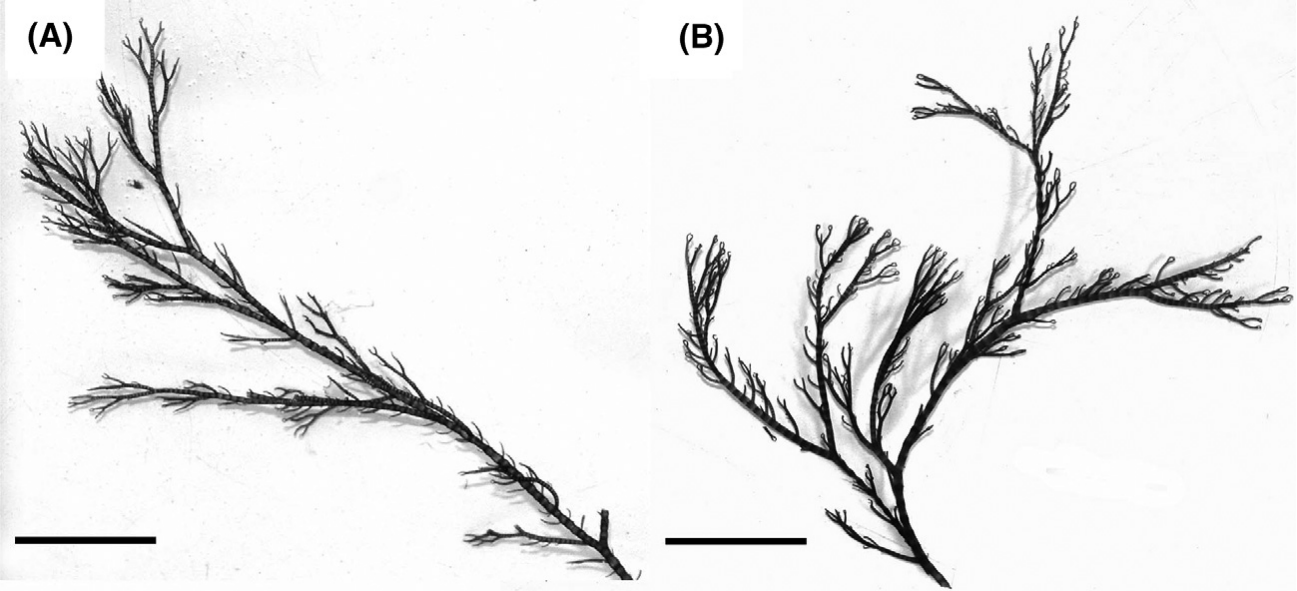
Shared morphological attributes in some individuals of *C. secundatum* and *C. virgatum*. (A) Gross morphology, cortication, and branching detail of *C. secundatum* (GWS030046). Scale bar = 1 cm. (B) Gross morphology, cortication, and branching detail of *C. virgatum* (GWS003615). Scale bar = 1 cm.

Without the aid of genetic analyses (COI‐5P and *rbc*L), *C. secundatum* would most likely have remained cryptic in the northwest Atlantic flora due to morphological attributes shared with *C. virgatum* (Fig. 2). Although these analyses were critical to the detection of *C. secundatum* in this flora, further analyses at the population level were required to ascertain whether this species was native or introduced. The genetic characteristics of a population arising from a species introduction are considered to resemble a founder event, in that in comparison with native populations the genetic variation is lower and haplotypes are subsets of those within the native range (Zink et al. 2000; Wares and Cunningham 2001). Our analyses of genetic variation indicated that *C. secundatum* in Narragansett Bay has a lower number of polymorphic sites, a lower average number of nucleotide differences (k), and lower nucleotide diversity (*π*) than populations within the native range (Table 1). These three measurements of genetic variation are consistent with *C. secundatum* being introduced to Narragansett Bay.

Notably, the population of *C. secundatum* in Narragansett Bay and the Dorn Lagoon population have a similar number of polymorphic sites, yet k and *π* were still almost 2× higher in the Dorn Lagoon population. Also, genetic variation within the Dorn Lagoon population was lower than the other populations within the native range (Dingle, Marble Hill, and Tramore). This may be an indication that *C. secundatum* was recently established at Dorn Lagoon, but was more likely a consequence of the limited area that specimens were found growing at this site, as a small population is more subject to genetic drift in the absence of gene flow (Ellstrand and Elam 1993).

**Figure 3:**
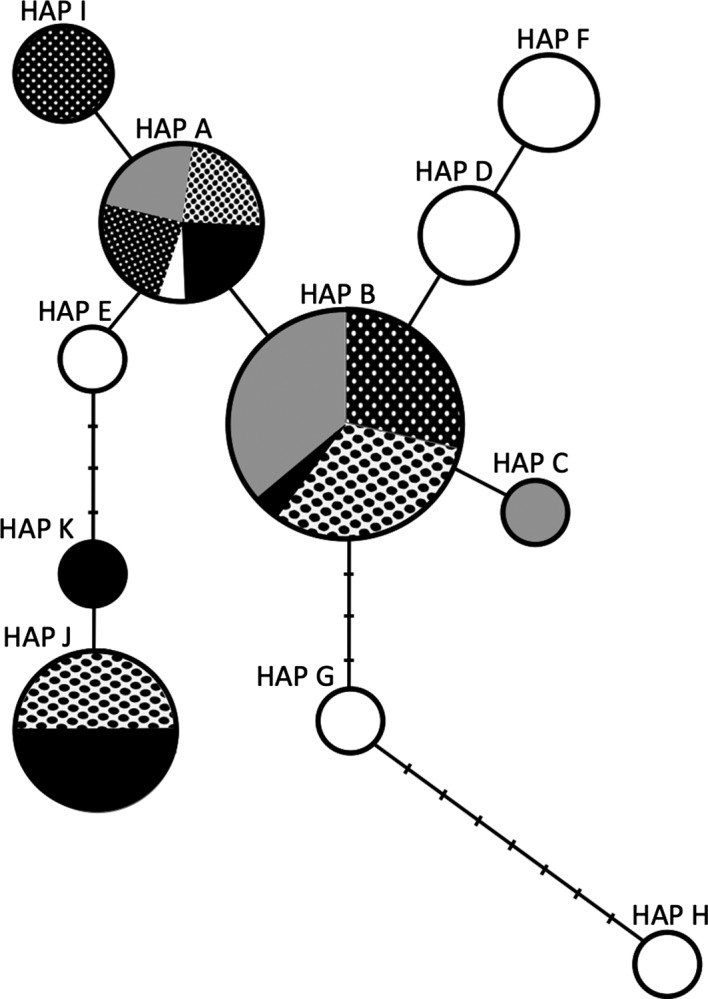
Haplotype network reconstructed using cox2‐3 spacer sequences obtained from *C. secundatum* specimens collected from Narragansett Bay (gray), Marble Hill (white), Dorn Lagoon (white dots), Tramore (black), and Dingle (black dots). The size of each circle (and partition within) is proportional to the relative frequency of each haplotype. Substitution differences between haplotypes are represented by a line, with multiple substitutions represented by marked points on the line.

We identified a total of eleven haplotypes (HAP A‐K; Fig. 3; Table 2) across all specimens included in this study. The most widespread haplotype, HAP A, was present in each of the populations. The second most widespread haplotype, HAP B, which was also the most common of the eleven haplotypes, represented 34% of specimens. Collectively, the most widespread and most abundant haplotypes (HAP A & HAP B) comprised the majority of specimens (93.8%) in the Narragansett Bay population. This observation was consistent with this population arising from an introduction.

Interestingly, there was one individual in the Narragansett Bay population that had a unique haplotype, HAP C, which was contrary to expectations for a population arising from an introduction. It is possible that HAP C is the result of a mutation within the Narragansett Bay population; however, rarefaction analyses (Table S3, Supporting information) estimated upwards of 51 haplotypes for the sampled region; and acknowledging that we collected specimens representing 11 of these haplotypes there is a considerable possibility that HAP C in our Narragansett Bay population represents an unsampled haplotype within the native range. Notably, we only sampled a small portion (four sites total in the Republic of Ireland and United Kingdom; Fig. 1) of *C. secundatum*'s natural range (Norway to Morocco), and rarefaction analyses do not make predictions for haplotypes beyond the sampled range. Therefore, it is likely that there are even more than 51 haplotypes for this species within its native range, which makes it more likely that HAP C was overlooked in the native range. The fact that each population had at least one unique haplotype, with the exception of Dingle, further emphasizes the probability that there are unsampled haplotypes within this species' native range.

From our comparisons of genetic variation and haplotypes within the *C. secundatum* population in Narragansett Bay to populations in the native range, we conclude that this species is not indigenous to the northwest Atlantic. Alternatively, a genetic bottleneck in a native North American *C. secundatum* population could yield similar patterns in genetic variation and haplotype diversity. Given that the only established population, we have found thus far is located near an active shipping port and that this species has been introduced to South Africa and New Zealand (C. Maggs, pers. comm.), we feel these circumstances favor the hypothesis that *C. secundatum* was introduced to this region.

How then, do we assess what the impacts of this specific introduction will be and determine whether or not it is likely to become invasive? In formulating predictions of the potential impacts of *C. secundatum* in the northwest Atlantic, we review what is known about the biology of this species relative to the new habitats. One weakness of this approach is that what is known about the biology of this species is based on observations within its native range and species may behave unpredictably when introduced to novel ecosystems (Lawrence et al. 2012). Nevertheless, introduced species that become invasive commonly share particular biological characteristics, such as broad environmental tolerances and asexual reproduction (Nyberg and Wallentinus 2005). Therefore, investigating the currently known biological characteristics of *C. secundatum* is a useful starting point in order to assess the potential impacts of this specific introduction.

In its native range, *C. secundatum* is often found in low intertidal pools or, more commonly, subtidal down to 11 m depth (Maggs and Hommersand 1993). It grows epiphytically (often on the introduced *Sargassum muticum* (Yendo) Fensholt, M. Bruce, pers. observation), epilithically, or on artificial materials in marinas (Maggs and Hommersand 1993). This species has been reported from sandy sheltered shallow bays, such as *Zostera marina* beds, as well as from sites with moderate wave exposure (Maggs and Hommersand 1993). Notably, not only is *C. secundatum* reported from a range of habitats, but these are indeed the predominant habitat types along the northwest Atlantic coast and would be the areas to experience the greatest impacts if *C. secundatum* was to naturalize and become invasive, in this region.

In considering reproduction, we did not find any reports of *C. secundatum* reproducing asexually, either through fragmentation or the production of various spore types; however, this does not rule out the possibility of asexual reproduction and this is something that should be considered in ongoing monitoring of this species in Narragansett Bay. With regard to hybridization, Maggs et al. (2002) eluded to the suspicion that *C. secundatum* may interbreed with *C. botrycarpum* A.W. Griffiths ex Harvey and that this relationship requires further investigation. However, *C. botrycarpum* is not reported in the northwest Atlantic flora. Considering that hybridization is more likely between of closely related taxa, and that *C. secundatum* is at least 10% divergent (COI‐5P) from all other northwest Atlantic *Ceramium* species identified during our ongoing DNA barcoding surveys (M. R. Bruce & G. W. Saunders, pers. observation), we do not regard *C. secundatum* as having the potential to hybridize with the native species and thus “genetic pollution” is not a concern.

The presently limited distribution of this species in the northwest Atlantic (Narragansett Bay and one drift specimen from Barnstable, Massachusetts) may reflect a recent introduction that has yet to spread, but could also be because *C. secundatum* is not an aggressive competitor with the native flora and has had a longer presence in the region. While it's reasonable to postulate the mode of introduction given the extensive shipping history of Narragansett Bay and the putative mode of other macroalgal introductions (Harlin and Villalard‐Bohnsack 2001; Schneider 2010), we currently cannot speculate on how or when *C. secundatum* was introduced.

It is important to note many introduced species that become invasive exhibit a lag phase before the full impacts of the introductions are observed, as was the case for *Grateloupia turuturu* Yamada in the northwest Atlantic and *Sargassum muticum* in the northeast Pacific (Lyons and Scheibling 2009). Only through the awareness of this introduction to the northwest Atlantic flora and continued biomonitoring will the impacts of *C. secundatum* in this region be fully elucidated.

## Conflict of Interest

None declared.

## Data accessibility

cox2‐3 spacer sequences: GenBank accessions (Table S1); NCBI SRA:
COI5‐P sequences: GenBank accessions (Table S1); NSBI SRA:
additional specimen information available in BOLD

## Supporting information


**Table S1.** Collection and GenBank information for specimens included in our DNA barcoding (COI‐5P) survey of northwest Atlantic *Ceramium* spp.
**Table S2.** *Ceramium secundatum* collections included in population genetic (cox2‐3 spacer) analyses and their respective GenBank accessions.
**Table S3.** Output from EstimateS analyses conducted in order to investigate the possibility of unsampled haplotypes within each of the four sites sampled within the native range of *C. secundatum*.
