# Supplementary material for: Population genetic analyses are consistent with the introduction of Ceramium secundatum (Ceramiaceae, Rhodophyta) to Narragansett Bay, Rhode Island, USA
Source: Ecol Evol. 2015 Oct 19;5(21):5088–95. doi: 10.1002/ece3.1754 (PMC4662316; doi:10.1002/ece3.1754)
Supplement: Supplementary file 1 — Table S1. Collection and GenBank information for specimens included in our DNA barcoding (COI‐5P) survey of northwest Atlantic Ceramium spp. Table S2. Ceramium secundatum collections included in population genetic (cox2‐3 spacer) analyses and their respective GenBank accessions. Table S3. Output from EstimateS analyses conducted in order to investigate the possibility of unsampled haplotypes within each of the four sites sampled within the native range of C. secundatum. [file ECE3-5-5088-s001.docx]

Supplementary Tables:

**Table S1** Collection and GenBank information for specimens included in our DNA barcoding (COI-5P) survey of northwest Atlantic *Ceramium* spp.

| Specimen # | *Genetic*  *identification* | Collection date | Collectors† | Lat | Long | GenBank accession # |
| --- | --- | --- | --- | --- | --- | --- |
| G0351 | *C. virgatum* | 08.10.1995 | GWS | 43.98899841 | -64.66200256 | KT250168 |
| GWS002355 | *C. virgatum* | 04.09.2004 | GWS | 45.24100113 | -64.35199738 | KT250192 |
| GWS002366 | *C. virgatum* | 05.09.2004 | GWS | 44.0870018 | -66.20300293 | KT250235 |
| GWS002662 | *C. virgatum* | 14.04.2005 | GWS | 43.56499863 | -70.1969986 | KT250250 |
| GWS003533 | *C. virgatum* | 14.11.2005 | GWS | 44.49000168 | -63.91699982 | KT250260 |
| GWS003543 | *C. virgatum* | 27.11.2005 | GWS | 44.625 | -66.86000061 | KT250247 |
| GWS003615 | *C. virgatum* | 24.04.2006 | LLG | 43.16600037 | -70.59200287 | KT250188 |
| GWS003647 | *C. virgatum* | 25.04.2006 | GWS, LLG, DM, SC & CL | 44.60900116 | -67.39700317 | HM918597 |
| GWS003742 | *C. virgatum* | 27.05.2006 | GWS | 44.625 | -66.86000061 | HM918603 |
| GWS003815 | *C. virgatum* | 28.05.2006 | GWS | 45.05599976 | -66.73600006 | KT250265 |
| GWS003816 | *C. virgatum* | 28.05.2006 | GWS | 45.05599976 | -66.73600006 | KT250193 |
| GWS005734 | *C. secundatum* | 10.08.2007 | GWS & BC | 41.47900009 | -71.36100006 | KT250218 |
| GWS005735 | *C. secundatum* | 10.08.2007 | GWS & BC | 41.47900009 | -71.36100006 | KT250270 |
| GWS005743 | *C. secundatum* | 10.08.2007 | GWS & BC | 41.47900009 | -71.36100006 | KT250236 |
| GWS005910 | *C. virgatum* | 19.03.2007 | GWS | 45.05599976 | -66.73600006 | KT250169 |
| GWS006087 | *C. secundatum* | 24.04.2007 | GWS, BC & DM | 41.47900009 | -71.361 | HM915257 |
| GWS006175 | *C. virgatum* | 15.05.2007 | GWS, DM & HK | 48.40000153 | -69.33899689 | HM916722 |
| GWS006223 | *C. virgatum* sp.2 | 27.08.2007 | GWS | 46.835 | -64.93 | KT250222 |
| GWS006236 | *C. virgatum* sp.2 | 27.08.2007 | GWS | 46.835 | -64.93 | KT250203 |
| GWS006967 | *C. virgatum* | 09.07.2006 | LLG, HK & JU | 45.97700119 | -60.79700089 | KT250219 |
| GWS006979 | *C. virgatum* | 10.07.2006 | LLG & JU | 47.6230011 | -59.29100037 | HM916855 |
| GWS007102 | *C. virgatum* | 12.07.2006 | LLG & JU | 49.52799988 | -59.29100037 | HM916868 |
| GWS007144 | *C. virgatum* | 13.07.2006 | LLG, HK & JU | 49.60699844 | -57.95000076 | HM916876 |
| GWS007179 | *C. virgatum* | 13.07.2006 | LLG, HK & JU | 49.60699844 | -57.95000076 | HM916882 |
| GWS007231 | *C. virgatum* | 14.07.2006 | LLG, BH & JU | 49.54800034 | -57.95399857 | HM916891 |
| GWS007418 | *C. virgatum* | 18.07.2006 | LLG, HK, DM & JU | 48.65499878 | -53.7519989 | HM916910 |
| GWS007423 | *C. virgatum* | 18.07.2006 | LLG, HK, DM & JU | 48.65499878 | -53.7519989 | HM916912 |
| GWS007454 | *C. virgatum* | 19.07.2006 | LLG, HK & DM | 48.65200043 | -53.90599823 | KT250249 |
| GWS007495 | *C. virgatum* | 20.07.2006 | LLG, DM & JU | 47.63199997 | -54.88600159 | HM916924 |
| GWS007507A | *C. virgatum* | 20.07.2006 | LLG, DM & JU | 47.63190079 | -54.88629913 | KT250221 |
| GWS007511 | *C. virgatum* | 20.07.2006 | LLG, DM & JU | 47.63199997 | -54.88600159 | HM916927 |
| GWS007522 | *C. virgatum* | 20.07.2006 | LLG, DM & JU | 47.63299942 | -54.86999893 | HM916929 |
| GWS007523 | *C. virgatum* | 20.07.2006 | LLG, DM & JU | 47.63299942 | -54.86999893 | HM916930 |
| GWS007686 | *C. virgatum* | 25.07.2006 | LLG, HK, DM & JU | 46.92100143 | -54.17399979 | HM916937 |
| GWS007701 | *C. virgatum* | 25.07.2006 | LLG, HK, DM & JU | 46.92100143 | -54.17399979 | KT250153 |
| GWS007714 | *C. virgatum* | 26.07.2006 | LLG, HK, DM & JU | 46.79899979 | -54.09799957 | HM916939 |
| GWS007757 | *C. virgatum* | 26.07.2006 | HK, DM & JU | 46.79899979 | -54.09799957 | KT250197 |
| GWS007762 | *C. virgatum* | 26.07.2006 | LLG, HK, DM & JU | 46.79899979 | -54.09799957 | KT250241 |
| GWS007792 | *C. virgatum* | 28.07.2006 | LLG & DM | 46.88199997 | -60.35100174 | HM916952 |
| GWS007833 | *C. virgatum* | 12.08.2006 | LLG & JU | 44.69200134 | -65.78500366 | HM916957 |
| GWS007837 | *C. virgatum* | 12.08.2006 | LLG & JU | 44.69200134 | -65.78500366 | HM916958 |
| GWS007872 | *C. virgatum* | 12.08.2006 | LLG & JU | 44.69200134 | -65.78500366 | HM916964 |
| GWS007894 | *C. virgatum* | 12.08.2006 | LLG & JU | 44.0870018 | -66.20300293 | HM916968 |
| GWS007975 | *C. virgatum* sp.2 | 17.08.2006 | LLG, HK & JU | 46.327 | -64.516 | HM916978 |
| GWS007991 | *C. virgatum* sp.2 | 17.08.2006 | LLG, HK & JU | 46.448 | -64.642 | HM916981 |
| GWS008008 | *C. virgatum* | 17.08.2006 | LLG, HK & JU | 46.95999908 | -64.83000183 | HM916985 |
| GWS008017 | *C. virgatum* | 17.08.2006 | LLG, HK & JU | 46.95999908 | -64.83000183 | HM916987 |
| GWS008793 | *C. virgatum* | 19.08.2007 | BC, KD & KR | 46.46300125 | -62.60699844 | KT250161 |
| GWS009337 | *C. virgatum* | 22.04.2008 | GWS, BC & DM | 48.40000153 | -69.33899689 | HM918914 |
| GWS009785 | *C. virgatum* | 30.05.2008 | HK & SH | 43.79000092 | -66.15499878 | HM917151 |
| GWS009786 | *C. virgatum* | 30.05.2008 | HK & SH | 43.79000092 | -66.15499878 | HM917152 |
| GWS009815 | *C. virgatum* | 31.05.2008 | HK & SH | 44.49000168 | -63.91699982 | HM917154 |
| GWS009817 | *C. virgatum* | 31.05.2008 | HK & SH | 44.49000168 | -63.91699982 | HM917155 |
| GWS009820 | *C. virgatum* | 31.05.2008 | HK & SH | 44.49000168 | -63.91699982 | HM917157 |
| GWS009833 | *C. virgatum* | 06.01.2008 | HK & SH | 45.64099884 | -61.7859993 | HM917159 |
| GWS009837 | *C. virgatum* | 06.01.2008 | HK & SH | 45.64099884 | -61.7859993 | HM917160 |
| GWS009857 | *C. virgatum* | 06.05.2008 | HK & SH | 45.0890007 | -61.69800186 | HM917162 |
| GWS009859 | *C. virgatum* | 06.05.2008 | HK & SH | 45.0890007 | -61.69800186 | HM917163 |
| GWS009876 | *C. virgatum* | 06.03.2008 | HK & SH | 44.70800018 | -62.8390007 | HM917166 |
| GWS009877 | *C. virgatum* | 06.03.2008 | HK & SH | 44.70800018 | -62.8390007 | HM917167 |
| GWS009882 | *C. virgatum* | 06.03.2008 | HK & SH | 44.70800018 | -62.8390007 | HM917169 |
| GWS009893 | *C. virgatum* | 03.09.2008 | GWS | 45.05599976 | -66.73600006 | HM917171 |
| GWS009894 | *C. virgatum* | 03.09.2008 | GWS | 45.05599976 | -66.73600006 | HM917172 |
| GWS011614A | *C. virgatum* | 28.07.2008 | GWS & MB | 46.49100113 | -63.30500031 | HM918331 |
| GWS011648 | *C. virgatum* | 28.07.2008 | GWS, DM, SH & MB | 46.45899963 | -63.27799988 | HM917354 |
| GWS011649 | *C. virgatum* | 28.07.2008 | GWS, DM, SH & MB | 46.45899963 | -63.27799988 | HQ919571 |
| GWS011668 | *C. virgatum* | 29.07.2008 | GWS, DM, SH & MB | 46.11399841 | -63.00600052 | HM917358 |
| GWS011682 | *C. virgatum* | 31.07.2008 | GWS, DM, SH & MB | 44.49000168 | -63.91699982 | HM917362 |
| GWS011684 | *C. virgatum* | 31.07.2008 | GWS, DM, SH & MB | 44.49000168 | -63.91699982 | HM917363 |
| GWS011703 | *C. virgatum* | 31.07.2008 | GWS, DM, SH & MB | 44.49000168 | -63.91699982 | HM917371 |
| GWS011705 | *C. virgatum* | 31.07.2008 | GWS, DM, SH & MB | 44.49000168 | -63.91699982 | HM917373 |
| GWS011738 | *C. virgatum* | 01.08.2008 | DM, MB & SH | 44.0870018 | -66.20300293 | HM917376 |
| GWS011741 | *C. virgatum* | 01.08.2008 | DM, MB & SH | 44.0870018 | -66.20300293 | HM917377 |
| GWS011836 | *C. virgatum* | 18.05.2009 | GWS & DM | 44.52600098 | -63.9469986 | HM915231 |
| GWS011869 | *C. virgatum* | 18.05.2009 | GWS & DM | 44.52600098 | -63.9469986 | HM915246 |
| GWS013888 | *C. virgatum* | 21.08.2009 | GWS & DM | 44.95700073 | -66.90399933 | HM916384 |
| GWS013928 | *C. virgatum* |  | AS | 46.83000183 | -64.91400146 | HQ919257 |
| GWS013980 | *C. virgatum* | 25.08.2010 | GWS, MB, AS & KD | 45.0379982 | -66.89099884 | HQ919498 |
| GWS013981 | *C. virgatum* | 25.08.2010 | GWS, MB, AS & KD | 45.0379982 | -66.89099884 | HQ919499 |
| GWS013982 | *C. virgatum* | 25.08.2010 | GWS, MB, AS & KD | 45.0379982 | -66.89099884 | HQ919500 |
| GWS013988 | *C. virgatum* | 25.08.2010 | GWS, MB, AS & KD | 45.0379982 | -66.89099884 | KT250164 |
| GWS014754 | *C. virgatum* | 12.04.2010 | BC, DM, MB, AS & CL | 41.93399811 | -70.55999756 | HM915056 |
| GWS014755 | *C. virgatum* | 13.04.2010 | DM, MB | 42.68500137 | -70.64099884 | HQ919214 |
| GWS014756 | *C. virgatum* | 13.04.2010 | DM, MB | 42.68500137 | -70.64099884 | KT250172 |
| GWS014783 | *C. virgatum* | 13.04.2010 | BC, AS | 42.68500137 | -70.64099884 | KT250266 |
| GWS014784 | *C. virgatum* | 13.04.2010 | BC & AS | 42.68500137 | -70.64099884 | KT250206 |
| GWS014785 | *C. virgatum* | 13.04.2010 | BC & AS | 42.68500137 | -70.64099884 | KT250148 |
| GWS014786 | *C. virgatum* | 13.04.2010 | BC & AS | 42.68500137 | -70.64099884 | KT250175 |
| GWS014787 | *C. virgatum* | 13.04.2010 | BC & AS | 42.68500137 | -70.64099884 | KT250239 |
| GWS014791 | *C. virgatum* | 13.04.2010 | BC & AS | 42.68500137 | -70.64099884 | KT250174 |
| GWS014792 | *C. virgatum* | 13.04.2010 | BC & AS | 42.68500137 | -70.64099884 | KT250227 |
| GWS017802 | *C. virgatum* | 13.04.2010 | BC & AS | 42.68500137 | -70.64099884 | KT250262 |
| GWS017935 | *C. virgatum* | 15.04.2010 | BC, DM, MB, AS & CL | 41.31999969 | -72.07499695 | KT250238 |
| GWS017987 | *C. secundatum* | 16.04.2010 | BC, DM, MB, AS & CL | 41.47900009 | -71.36100006 | KT250146 |
| GWS017988 | *C. secundatum* | 16.04.2010 | BC, DM, MB, AS & CL | 41.47900009 | -71.36100006 | KT250210 |
| GWS017989 | *C. secundatum* | 16.04.2010 | BC, DM, MB, AS & CL | 41.47900009 | -71.36100006 | KT250205 |
| GWS017990 | *C. secundatum* | 16.04.2010 | BC, DM, MB, AS & CL | 41.47900009 | -71.36100006 | KT250184 |
| GWS018001 | *C. secundatum* | 16.04.2010 | BC, DM, MB, AS & CL | 41.47900009 | -71.36100006 | KT250214 |
| GWS018002 | *C. secundatum* | 16.04.2010 | BC, DM, MB, AS & CL | 41.47900009 | -71.36100006 | KT250216 |
| GWS018003 | *C. secundatum* | 16.04.2010 | BC, DM, MB, AS & CL | 41.47900009 | -71.36100006 | KT250196 |
| GWS018004 | *C. secundatum* | 16.04.2010 | BC, DM, MB, AS & CL | 41.47900009 | -71.36100006 | KT250231 |
| GWS018024 | *C. secundatum* | 16.04.2010 | BC, DM, MB, AS & CL | 41.47900009 | -71.36100006 | KT250166 |
| GWS018025 | *C. secundatum* | 16.04.2010 | BC, DM, MB, AS & CL | 41.47900009 | -71.36100006 | KT250186 |
| GWS018033 | *C. secundatum* | 16.04.2010 | BC, DM, MB, AS & CL | 41.47900009 | -71.36100006 | KT250217 |
| GWS018036 | *C. secundatum* | 16.04.2010 | BC, DM, MB, AS & CL | 41.47900009 | -71.36100006 | KT250150 |
| GWS018041†† | *C. secundatum* | 16.04.2010 | BC, DM, MB, AS & CL | 41.47900009 | -71.36100006 | KT250208 |
| GWS018042 | *C. secundatum* | 16.04.2010 | BC, DM, MB, AS & CL | 41.47900009 | -71.36100006 | KT250190 |
| GWS018043 | *C. secundatum* | 16.04.2010 | BC, DM, MB, AS & CL | 41.47900009 | -71.36100006 | KT250237 |
| GWS018055 | *C. secundatum* | 16.04.2010 | BC, DM, MB, AS & CL | 41.47900009 | -71.36100006 | KT250242 |
| GWS018089 | *C. virgatum* | 19.04.2010 | BC, DM, MB, AS & CL | 43.56499863 | -70.1989975 | KT250259 |
| GWS018093 | *C. virgatum* | 19.04.2010 | BC, DM, MB, AS & CL | 43.56499863 | -70.1989975 | KT250189 |
| GWS018094 | *C. virgatum* | 19.04.2010 | BC, DM, MB, AS & CL | 43.56499863 | -70.1989975 | KT250226 |
| GWS018095 | *C. virgatum* | 19.04.2010 | BC, DM, MB, AS & CL | 43.56499863 | -70.1989975 | KT250155 |
| GWS018096 | *C. virgatum* | 19.04.2010 | BC, DM, MB, AS & CL | 43.56499863 | -70.1989975 | KT250215 |
| GWS018099 | *C. virgatum* | 19.04.2010 | BC, DM, MB, AS & CL | 43.56499863 | -70.1989975 | KT250207 |
| GWS018101 | *C. virgatum* | 19.04.2010 | BC, DM, MB, AS & CL | 43.56499863 | -70.1989975 | KT250152 |
| GWS018102 | *C. virgatum* | 19.04.2010 | BC, DM, MB, AS & CL | 43.56499863 | -70.1989975 | KT250202 |
| GWS018103 | *C. virgatum* | 19.04.2010 | BC, DM, MB, AS & CL | 43.56499863 | -70.1989975 | KT250246 |
| GWS018105 | *C. virgatum* | 19.04.2010 | BC, DM, MB, AS & CL | 43.56499863 | -70.1989975 | KT250187 |
| GWS018106 | *C. virgatum* | 19.04.2010 | BC, DM, MB, AS & CL | 43.56499863 | -70.1989975 | KT250263 |
| GWS018107 | *C. virgatum* | 19.04.2010 | BC, DM, MB, AS & CL | 43.56499863 | -70.1989975 | KT250185 |
| GWS018109 | *C. virgatum* | 19.04.2010 | BC, DM, MB, AS & CL | 43.56499863 | -70.1989975 | KT250204 |
| GWS018110 | *C. virgatum* | 19.04.2010 | BC, DM, MB, AS & CL | 43.56499863 | -70.1989975 | KT250209 |
| GWS018111 | *C. virgatum* | 19.04.2010 | BC, DM, MB, AS & CL | 43.56499863 | -70.1989975 | KT250243 |
| GWS018113 | *C. virgatum* | 19.04.2010 | BC, DM, MB, AS & CL | 43.56499863 | -70.1989975 | KT250198 |
| GWS018114 | *C. virgatum* | 19.04.2010 | BC, DM, MB, AS & CL | 43.56499863 | -70.1989975 | KT250213 |
| GWS018117 | *C. virgatum* | 19.04.2010 | BC, DM, MB, AS & CL | 43.56499863 | -70.1989975 | KT250154 |
| GWS018119 | *C. virgatum* | 19.04.2010 | BC, DM, MB, AS & CL | 43.56499863 | -70.1989975 | KT250195 |
| GWS018120 | *C. virgatum* | 19.04.2010 | BC, DM, MB, AS & CL | 43.56499863 | -70.1989975 | KT250261 |
| GWS018121 | *C. virgatum* | 19.04.2010 | BC, DM, MB, AS & CL | 43.56499863 | -70.1989975 | KT250253 |
| GWS018122 | *C. virgatum* | 19.04.2010 | BC, DM, MB, AS & CL | 43.56499863 | -70.1989975 | KT250160 |
| GWS018124 | *C. virgatum* | 19.04.2010 | BC, DM, MB, AS & CL | 43.56499863 | -70.1989975 | KT250177 |
| GWS018126 | *C. virgatum* | 19.04.2010 | BC, DM, MB, AS & CL | 43.56499863 | -70.1989975 | KT250183 |
| GWS018129 | *C. virgatum* | 19.04.2010 | BC, DM, MB, AS & CL | 43.56499863 | -70.1989975 | KT250257 |
| GWS018134 | *C. virgatum* | 19.04.2010 | BC, DM, MB, AS & CL | 43.56499863 | -70.1989975 | KT250264 |
| GWS018137 | *C. virgatum* | 19.04.2010 | BC, DM, MB, AS, CL | 43.56499863 | -70.1989975 | KT250232 |
| GWS018139 | *C. virgatum* | 19.04.2010 | BC, DM, MB, AS, CL | 43.56499863 | -70.1989975 | KT250170 |
| GWS027604 | *C. virgatum* | 26.07.2011 | MB & AR | 46.63899994 | -60.38999939 | KT250181 |
| GWS027605 | *C. virgatum* | 26.07.2011 | MB & AR | 46.63899994 | -60.38999939 | KT250263 |
| GWS027606 | *C. virgatum* | 26.07.2011 | MB & AR | 46.63899994 | -60.38999939 | KT250268 |
| GWS027607 | *C. virgatum* | 26.07.2011 | MB & AR | 46.63899994 | -60.38999939 | KT250159 |
| GWS027608 | *C. virgatum* | 26.07.2011 | MB & AR | 46.63899994 | -60.38999939 | KT250199 |
| GWS027609 | *C. virgatum* | 26.07.2011 | MB & AR | 46.63899994 | -60.38999939 | KT250271 |
| GWS027611 | *C. virgatum* | 26.07.2011 | MB & AR | 46.63899994 | -60.38999939 | KT250254 |
| GWS027612 | *C. virgatum* | 26.07.2011 | MB & AR | 46.63899994 | -60.38999939 | KT250182 |
| GWS027613 | *C. virgatum* | 26.07.2011 | MB & AR | 46.63899994 | -60.38999939 | KT250229 |
| GWS027614 | *C. virgatum* | 26.07.2011 | MB & AR | 46.63899994 | -60.38999939 | KT250167 |
| GWS027615 | *C. virgatum* | 26.07.2011 | MB & AR | 46.63899994 | -60.38999939 | KT250180 |
| GWS027616 | *C. virgatum* | 26.07.2011 | MB & AR | 46.63899994 | -60.38999939 | KT250223 |
| GWS027803 | *C. virgatum* | 13.04.2011 | GWS, KD, AS, MB & DM | 41.7519989 | -70.18699646 | KT250147 |
| GWS027804 | *C. secundatum* | 13.04.2011 | GWS, KD, AS, MB & DM | 41.7519989 | -70.18699646 | KT250269 |
| GWS027805 | *C. virgatum* | 13.04.2011 | GWS, KD, AS, MB & DM | 41.7519989 | -70.18699646 | KT250224 |
| GWS027806 | *C. virgatum* | 13.04.2011 | GWS, KD, AS, MB & DM | 41.7519989 | -70.18699646 | KT250201 |
| GWS030043 | *C. secundatum* | 17.04.2012 | MB & KD | 41.47900009 | -71.36100006 | KT250156 |
| GWS030046 | *C. secundatum* | 17.04.2012 | MB & KD | 41.47900009 | -71.36100006 | KT250194 |
| GWS030047 | *C. secundatum* | 17.04.2012 | MB & KD | 41.47900009 | -71.36100006 | KT250158 |
| GWS030073 | *C. virgatum* | 17.04.2012 | GWS, AS, MB & KD | 41.41400146 | -71.45300293 | KT250267 |
| GWS030074 | *C. virgatum* | 17.04.2012 | GWS, AS, MB & KD | 41.41400146 | -71.45300293 | KT250240 |
| GWS030075 | *C. virgatum* | 17.04.2012 | GWS, AS, MB & KD | 41.41400146 | -71.45300293 | KT250179 |
| GWS030078 | *C. virgatum* | 17.04.2012 | GWS, AS, MB & KD | 41.41400146 | -71.45300293 | KT250225 |
| GWS030080 | *C. virgatum* | 17.04.2012 | GWS, AS, MB & KD | 41.41400146 | -71.45300293 | KT250176 |
| GWS030082 | *C. secundatum* | 17.04.2012 | GWS, AS, MB & KD | 41.41400146 | -71.45300293 | KT250245 |
| GWS030083 | *C. virgatum* | 17.04.2012 | GWS, AS, MB & KD | 41.41400146 | -71.45300293 | KT250145 |
| GWS030084 | *C. virgatum* | 17.04.2012 | GWS, AS, MB & KD | 41.41400146 | -71.45300293 | KT250258 |
| GWS030085 | *C. virgatum* | 17.04.2012 | GWS, AS, MB & KD | 41.41400146 | -71.45300293 | KT250220 |
| GWS030086 | *C. virgatum* | 17.04.2012 | GWS, AS, MB & KD | 41.41400146 | -71.45300293 | KT250228 |
| GWS030089 | *C. virgatum* | 17.04.2012 | GWS, AS, MB & KD | 41.41400146 | -71.45300293 | KT250200 |
| GWS030090 | *C. virgatum* | 17.04.2012 | GWS, AS, MB & KD | 41.41400146 | -71.45300293 | KT250162 |
| GWS030091 | *C. virgatum* | 17.04.2012 | GWS, AS, MB & KD | 41.41400146 | -71.45300293 | KT250251 |
| GWS030092 | *C. virgatum* | 17.04.2012 | GWS, AS, MB & KD | 41.42200089 | -71.45500183 | KT250244 |
| GWS030093 | *C. virgatum* | 17.04.2012 | GWS, AS, MB & KD | 41.42200089 | -71.45500183 | KT250234 |
| GWS030094 | *C. virgatum* | 17.04.2012 | GWS, AS, MB & KD | 41.42200089 | -71.45500183 | KT250255 |
| GWS030095 | *C. secundatum* | 17.04.2012 | GWS, AS, MB & KD | 41.42200089 | -71.45500183 | KT250211 |
| GWS030096 | *C. virgatum* | 17.04.2012 | GWS, AS, MB & KD | 41.42200089 | -71.45500183 | KT250230 |
| GWS030097 | *C. virgatum* | 17.04.2012 | GWS, AS, MB & KD | 41.42200089 | -71.45500183 | KT250212 |
| GWS030098 | *C. virgatum* | 17.04.2012 | GWS, AS, MB & KD | 41.42200089 | -71.45500183 | KT250191 |
| GWS030149 | *C. virgatum* | 19.04.2012 | GWS | 42.68500137 | -70.64099884 | KT250171 |
| GWS030150 | *C. virgatum* | 19.04.2012 | GWS | 42.68500137 | -70.64099884 | KT250248 |
| GWS030156 | *C. virgatum* | 19.04.2012 | MB | 42.68500137 | -70.64099884 | KT250173 |
| GWS030164 | *C. virgatum* | 19.04.2012 | AS | 42.68500137 | -70.64099884 | KT250165 |
| GWS030167 | *C. virgatum* | 19.04.2012 | AS | 42.68500137 | -70.64099884 | KT250163 |
| GWS030177 | *C. virgatum* | 20.04.2012 | MB | 44.33200073 | -68.06199646 | KT250252 |
| GWS030219 | *C. virgatum* | 15.05.2012 | GWS, AS, MB & KD | 45.13000107 | -66.52600098 | KT250178 |
| GWS030247 | *C. virgatum* | 15.05.2012 | GWS, AS, MB & KD | 45.13000107 | -66.52600098 | KT250157 |
| GWS030250 | *C. virgatum* | 15.05.2012 | GWS, AS, MB & KD | 45.13000107 | -66.52600098 | KT250256 |
| GWS031539 | *C. virgatum* | 04.07.2012 | GWS, AS & GF | 45.03900146 | -66.80799866 | KT250151 |
| GWS032010 | *C. virgatum* | 13.08.2012 | GWS & AS | 44.50799942 | -64.12599945 | KT250233 |
| GWS032303 | *C. virgatum* | 11.09.2012 | MB | 45.10900116 | -66.48200226 | KT250149 |

† AR= Alex Ready; AS= Amanda Savoie; BC= Bridgette Clarkston; BH= Bob Hooper; DM= Dan McDevit; CL= Caroline Longtin; CAM= Christine Maggs; GWS= Gary Saunders; GF= Gina Filloramo; HK = Hana Kucera; JU= Jose Utge; KD= Kyatt Dixon; KR= Kathryn Roy; LLG= Line LeGall; MB= Meghann Bruce; SH= Sarah Hamsher; and, SC= Susan Clayden.

**Table S2** *Ceramium secundatum* collections included in population genetic (cox2-3 spacer) analyses and their respective GenBank accessions.

| Collection location | Specimen # | Collection date | Collectors† | GenBank accession # |
| --- | --- | --- | --- | --- |
| Narragansett Bay,  Rhode Island, USA  41.47910, -7136066 | GWS006087 | 24.4.2007 | GWS, BC & DM | KT250346 |
|  | GWS017988 | 16.4.2010 | BC, DM, MB, AS & CL | KT250317 |
|  | GWS017989 | 16.4.2010 | BC, DM, MB, AS & CL | KT250312 |
|  | GWS017990 | 16.4.2010 | BC, DM, MB, AS & CL | KT250299 |
|  | GWS018001 | 16.4.2010 | BC, DM, MB, AS & CL | KT250318 |
|  | GWS018002 | 16.4.2010 | BC, DM, MB, AS & CL | KT250321 |
|  | GWS018003 | 16.4.2010 | BC, DM, MB, AS & CL | KT250308 |
|  | GWS018004 | 16.4.2010 | BC, DM, MB, AS & CL | KT250332 |
|  | GWS018024 | 16.4.2010 | BC, DM, MB, AS & CL | KT250288 |
|  | GWS018025 | 16.4.2010 | BC, DM, MB, AS & CL | KT250301 |
|  | GWS018033 | 16.4.2010 | BC, DM, MB, AS & CL | KT250322 |
|  | GWS018036 | 16.4.2010 | BC, DM, MB, AS & CL | KT250276 |
|  | GWS018041 | 16.4.2010 | BC, DM, MB, AS & CL | KT250315 |
|  | GWS018042 | 16.4.2010 | BC, DM, MB, AS & CL | KT250305 |
|  | GWS018043 | 16.4.2010 | BC, DM, MB, AS & CL | KT250342 |
|  | GWS018055 | 16.4.2010 | BC, DM, MB, AS & CL | KT250344 |
| Marble Hill, Republic of Ireland  55.17634,-7.89878 | GWS031587 | 22.7.2012 | MB | KT250350 |
|  | GWS031588 | 22.7.2012 | MB | KT250281 |
|  | GWS031589 | 22.7.2012 | MB | KT250335 |
|  | GWS031590 | 22.7.2012 | MB | KT250350 |
|  | GWS031592 | 22.7.2012 | MB | KT250281 |
|  | GWS031593 | 22.7.2012 | MB | KT250335 |
|  | GWS031594 | 22.7.2012 | MB | KT250325 |
|  | GWS031595 | 22.7.2012 | MB | KT250339 |
|  | GWS031596 | 22.7.2012 | MB | KT250329 |
|  | GWS031598 | 22.7.2012 | MB | KT250278 |
|  | GWS031823 | 22.7.2012 | MB | KT250319 |
|  | GWS031824 | 22.7.2012 | MB | KT250300 |
|  | GWS031826 | 22.7.2012 | MB | KT250287 |
|  | GWS031827 | 22.7.2012 | MB | KT250359 |
|  | GWS031828 | 22.7.2012 | MB | KT250348 |
|  | GWS031830 | 22.7.2012 | MB | KT250290 |
|  | GWS031831 | 22.7.2012 | MB | KT250364 |
|  | GWS031833 | 22.7.2012 | MB | KT250334 |
|  | GWS031834 | 22.7.2012 | MB | KT250349 |
|  | GWS031835 | 22.7.2012 | MB | KT250330 |
| Dorn Lagoon, Northern Ireland, U.K.  54.43376, -5.53412 | GWS031837 | 1.7.2012 | MB | KT250309 |
|  | GWS031839 | 1.7.2012 | CAM & MB | KT250274 |
|  | GWS031840 | 1.7.2012 | CAM & MB | KT250285 |
|  | GWS031842 | 1.7.2012 | CAM & MB | KT250297 |
|  | GWS031843 | 1.7.2012 | CAM & MB | KT250316 |
|  | GWS031844 | 1.7.2012 | CAM & MB | KT250340 |
|  | GWS031882 | 9.7.2012 | MB & AF | KT250352 |
|  | GWS031883 | 9.7.2012 | MB & AF | KT250331 |
|  | GWS031885 | 9.7.2012 | MB & AF | KT250296 |
|  | GWS031887 | 9.7.2012 | MB & AF | KT250284 |
|  | GWS031889 | 9.7.2012 | MB & AF | KT250282 |
|  | GWS031892 | 9.7.2012 | MB & AF | KT250328 |
|  | GWS031893 | 9.7.2012 | MB & AF | KT250327 |
|  | GWS031894 | 9.7.2012 | MB & AF | KT250362 |
|  | GWS031897 | 9.7.2012 | MB & AF | KT250338 |
|  | GWS031898 | 9.7.2012 | MB & AF | KT250279 |
|  | GWS031899 | 9.7.2012 | MB & AF | KT250353 |
|  | GWS031900 | 9.7.2012 | MB & AF | KT250277 |
| Dingle, Republic of Ireland  52.13854, -10.2723 | GWS031941 | 16.7.2012 | MB & AF | KT250304 |
|  | GWS031942 | 16.7.2012 | MB & AF | KT250302 |
|  | GWS031943 | 16.7.2012 | MB & AF | KT250326 |
|  | GWS031944 | 16.7.2012 | MB & AF | KT250314 |
|  | GWS031945 | 16.7.2012 | MB & AF | KT250360 |
|  | GWS031947 | 16.7.2012 | MB & AF | KT250351 |
|  | GWS031948 | 16.7.2012 | MB & AF | KT250361 |
|  | GWS031949 | 16.7.2012 | MB & AF | KT250313 |
|  | GWS031952 | 16.7.2012 | MB & AF | KT250303 |
|  | GWS031953 | 16.7.2012 | MB & AF | KT250311 |
|  | GWS031957 | 16.7.2012 | MB & AF | KT250345 |
|  | GWS031958 | 16.7.2012 | MB & AF | KT250354 |
|  | GWS031959 | 16.7.2012 | MB & AF | KT250275 |
|  | GWS031960 | 16.7.2012 | MB & AF | KT250280 |
|  | GWS031961 | 16.7.2012 | MB & AF | KT250295 |
|  | GWS031962 | 16.7.2012 | MB & AF | KT250306 |
|  | GWS031963 | 16.7.2012 | MB & AF | KT250289 |
|  | GWS031964 | 16.7.2012 | MB & AF | KT250341 |
|  | GWS031965 | 16.7.2012 | MB & AF | KT250343 |
|  | GWS031966 | 16.7.2012 | MB & AF | KT250294 |
|  | GWS031967 | 16.7.2012 | MB & AF | KT250283 |
|  | GWS031969 | 16.7.2012 | MB & AF | KT250292 |
| Tramore, Republic of  Ireland  52.15413, -7.12085 | GWS031909 | 13.7.2012 | MB & AF | KT250336 |
|  | GWS031912 | 13.7.2012 | MB & AF | KT250358 |
|  | GWS031914 | 13.7.2012 | MB & AF | KT250355 |
|  | GWS031915 | 13.7.2012 | MB & AF | KT250323 |
|  | GWS031916 | 13.7.2012 | MB & AF | KT250337 |
|  | GWS031918 | 13.7.2012 | MB & AF | KT250298 |
|  | GWS031919 | 13.7.2012 | MB & AF | KT250357 |
|  | GWS031922 | 13.7.2012 | MB & AF | KT250291 |
|  | GWS031925 | 13.7.2012 | MB & AF | KT250324 |
|  | GWS031928 | 13.7.2012 | MB & AF | KT250356 |
|  | GWS031929 | 13.7.2012 | MB & AF | KT250310 |
|  | GWS031931 | 13.7.2012 | MB & AF | KT250363 |
|  | GWS031932 | 13.7.2012 | MB & AF | KT250320 |
|  | GWS031934 | 13.7.2012 | MB & AF | KT250286 |
|  | GWS031935 | 13.7.2012 | MB & AF | KT250347 |

† AF = Aaron Frenette; AS= Amanda Savoie; BC= Bridgette Clarkston; DM= Dan McDevit; CL= Caroline Longtin; CAM= Christine Maggs; GWS= Gary Saunders; KD= Kyatt Dixon; and MB= Meghann Bruce.

**Table S3** Output from EstimateS analyses conducted in order to investigate the possibility of unsampled haplotypes within each of the four sites sampled within the native range of *C. secundatum*.

| Samples | Individuals (computed) | Sobs (Mao Tau) | Sobs 95% CI Lower Bound |
| --- | --- | --- | --- |
| 1 | 18.75 | 4 | 1.44 |
| 2 | 37.5 | 6.33 | 2.89 |
| 3 | 56.25 | 8.25 | 4.04 |
| 4 | 75 | 10 | 4.97 |
|  |  |  |  |
|  |  |  |  |
| Samples | Sobs 95% CI Upper Bound | Sobs SD (Mao Tau) | Sobs Mean (runs) |
| 1 | 6.56 | 1.31 | 3.74 |
| 2 | 9.77 | 1.75 | 6.26 |
| 3 | 12.46 | 2.15 | 8.66 |
| 4 | 15.03 | 2.57 | 10 |
|  |  |  |  |
|  |  |  |  |
| Samples | Singletons Mean | Singletons SD (runs) | Doubletons Mean |
| 1 | 1.12 | 1.57 | 0 |
| 2 | 2.08 | 1.58 | 0 |
| 3 | 3.3 | 1.09 | 0 |
| 4 | 4 | 0 | 0 |
|  |  |  |  |
|  |  |  |  |
| Samples | Doubletons SD (runs) | Uniques Mean | Uniques SD (runs) |
| 1 | 0 | 3.74 | 1.14 |
| 2 | 0 | 4.56 | 2.79 |
| 3 | 0 | 6.18 | 2.01 |
| 4 | 0 | 7 | 0 |
|  |  |  |  |
|  |  |  |  |
| Samples | Duplicates Mean | Duplicates SD (runs) | ACE Mean |
| 1 | 0 | 0 | 5.6 |
| 2 | 1.7 | 0.79 | 8.6 |
| 3 | 1.32 | 0.47 | 13.04 |
| 4 | 1 | 0 | 16 |
|  |  |  |  |
|  |  |  |  |
| Samples | ACE SD (runs) | ICE Mean | ICE SD (runs) |
| 1 | 3.59 | 8.06 | 4.53 |
| 2 | 5.01 | 35.86 | 29.38 |
| 3 | 3.84 | 28.99 | 12.95 |
| 4 | 0 | 28.38 | 0 |
|  |  |  |  |
|  |  |  |  |
| Samples | Chao 1 Mean | Chao 1 95% CI Lower Bound | Chao 1 95% CI Upper Bound |
| 1 | 5.02 | 3.93 | 13.17 |
| 2 | 8.6 | 6.63 | 22.01 |
| 3 | 13.04 | 9.33 | 37.58 |
| 4 | 16 | 10.95 | 47.82 |
|  |  |  |  |
|  |  |  |  |
| Samples | Chao 1 SD (analytical) | Chao 2 Mean | Chao 2 95% CI Lower Bound |
| 1 | 1.74 | 8.06 | 4.51 |
| 2 | 3.06 | 10.66 | 7.1 |
| 3 | 5.48 | 14.8 | 9.84 |
| 4 | 7.14 | 17.88 | 11.5 |
|  |  |  |  |
|  |  |  |  |
| Samples | Chao 2 95% CI Upper Bound | Chao 2 SD (analytical) | Jack 1 Mean |
| 1 | 29.7 | 4.9 | 3.74 |
| 2 | 30.61 | 4.61 | 8.54 |
| 3 | 41.31 | 6.32 | 12.78 |
| 4 | 51.39 | 8.06 | 15.25 |
|  |  |  |  |
|  |  |  |  |
| Samples | Jack 1 SD (analytical) | Jack 2 Mean | Jack 2 SD (runs) |
| 1 | 0 | 0 | 0 |
| 2 | 0.86 | 8.54 | 3.44 |
| 3 | 2.39 | 14.62 | 3.66 |
| 4 | 3.33 | 18.42 | 0 |
|  |  |  |  |
|  |  |  |  |
| Samples | Bootstrap Mean | Bootstrap SD (runs) | MMRuns Mean |
| 1 | 3.74 | 1.14 | 0 |
| 2 | 7.4 | 2.74 | 8.81 |
| 3 | 10.54 | 2.26 | 16.31 |
| 4 | 12.28 | 0 | 46.32 |
|  |  |  |  |
|  |  |  |  |
| Samples | MMMeans (1 run) | Cole Rarefaction | Cole SD (analytical) |
| 1 | 0 | 6.53 | 1.07 |
| 2 | 15.2 | 7.96 | 1.02 |
| 3 | 17.28 | 9 | 0.87 |
| 4 | 19.37 |  |  |
|  |  |  |  |
|  |  |  |  |
| Samples | Alpha Mean | Alpha SD (analytical) | Shannon Mean |
| 1 | 0 | 0 | 0 |
| 2 | 0 | 0 | 0 |
| 3 | 0 | 0 | 0 |
| 4 | 0 | 0 | 0 |
|  |  |  |  |
|  |  |  |  |
| Samples | Shannon SD (runs) | Simpson Mean | Simpson SD (runs) |
| 1 | 0 | 0 | 0 |
| 2 | 0 | 0 | 0 |
| 3 | 0 | 0 | 0 |
| 4 | 0 | 0 | 0 |
|  |  |  |  |
|  |  |  |  |
